# Does social capital buffer or exacerbate mental health inequality? Evidence from the China Family Panel Study (CFPS)

**DOI:** 10.1186/s12939-022-01642-3

**Published:** 2022-05-23

**Authors:** Dan Cao, Zhongliang Zhou, Guanping Liu, Chi Shen, Yangling Ren, Dantong Zhao, Yaxin Zhao, Qiwei Deng, Xiaohui Zhai

**Affiliations:** 1 School of Public Policy and Administration, Xi’an Jiaotong University, No. 28 Xianning West Road, Xi’an, Shaanxi PR China; 2 School of Public Health, Xi’an Jiaotong University, Xi’an, PR China; 3 School of Public Administration, Southwestern University of Finance and Economics, Xi’an, PR China

**Keywords:** Social capital, Mental health, Inequality, Depressive symptoms, Subjective well-being

## Abstract

**Background:**

Health inequality, including physical and mental health inequality, is an important issue. What role social capital plays in mental health inequality is still ambiguous, especially in developing countries. The aim of this study is to explore the relationship between social capital and mental health inequality in China.

**Method:**

Both family-level and community-/village-level social capitals are included in our analysis. Data is mainly extracted from the China Family Panel Studies in 2018, and lagged term of social capital in CFPS 2016 was used to link with other variables in 2018. Depressive symptoms and subjective well-being are set as indicators of mental health. A series of OLS regression models were conducted to estimate the effects of social capital on mental health and mental health inequality.

**Results:**

Higher levels of social capital and income are related to a lower level of depressive symptoms and a higher level of subjective well-being. The positive coefficient of interaction term of family-level social capital and income level in the urban area indicates that the inhibiting effect of social capital on depressive symptoms is pro-poor. The negative coefficient of interaction term of village-level social capital and income level in the rural area suggests that the promoting effect of social capital on subjective well-being is pro-poor, too.

**Conclusion:**

The results show that severe mental health inequality exists in China; family-level social capital can buffer depressive symptom inequality, and village-level social capital can buffer SWB inequality. Although the amount of social capital of the poor is less than the rich, the poor can better use social capital to improve their mental health. Our study advocates enhancing social participation and communication for the poor to reduce mental health inequality.

**Supplementary Information:**

The online version contains supplementary material available at 10.1186/s12939-022-01642-3.

## Introduction

Income-related health inequality has been one of the hottest topics in research fields of health economics and public health, and has arose the intense interest of health policymakers. Health inequality exists all over the world, especially in low- and middle-income countries such as China [1–3]. It is widely acknowledged that better health is associated with a higher level of income, educational status, socioeconomic status (SES), and more advantaged ethnic groups. A study using nationally representative data found that although average per capita income had increased a lot in the past decades in China, income-related health inequality had also doubled [4]. Another study focusing on health outcomes and SES suggested that higher SES could positively affect health indicators such as hypertension situation and life-functioning among the Chinese elderly [5]. Not only physical health but also mental health is severely unequal in China. Previous research using data from the China Health and Retirement Longitudinal Study found that for mid-aged and elderly, the individual depressive symptom was significantly related to personal educational status and expenditure [6]. Inequality caused by the *hukou* system also arises mental health inequality. Scholars found that urban residents possessed a lower level of depressive symptoms than rural residents and temporary migrants [7]. It is apparent that health inequality, including physical and mental health inequality, is an important issue in current China.

Since *social capital (SC)* was brought forward by Bourdieu [8], it has gained greatly developed by some other scholars such as Coleman [9] and Putnam [10]. The original definition of social capital is “aggregate of the actual or potential resources which are linked to possession of a durable network of more or less institutionalized”, given by Bourdieu [8]. Putnam defined SC as “features of social organization, such as trust, norms, and networks that can improve the efficiency of society by facilitating coordinated actions” [10], which was widely accepted by other scholars. Different approaches to measuring social capital are adopted in empirical studies. A common distinction is structural and cognitive dimensions. Structural social capital refers to the individual’s participation in formal associations such as religious organizations and labor unions, neighborhood activities, and social resources from these associations and activities. The cognitive dimension describes an individual’s attitude, such as trust and social cohesion of the neighborhood. Yip and colleagues measured structural social capital by how many organizations residents participated in, cognitive social capital by scores related to trust, reciprocity, and mutual help [11]. Another distinction often used is bonding, bridging, and linking components, while the bonding dimension is defined as the relationship with relatives or friends, the bridging dimension is measured by the relationship between more loosely connected people such as neighbors or colleagues, the linking dimension refers to relationship connected by authority or power such as employer and employee [12]. In health research fields, social capital has long been regarded as a useful intervention to promote an individual’s health status. For example, using nationally representative data and measuring social capital by social participation, Liu and colleagues found that SC had a significantly positive effect on the elderly’s physical health and well-being [13]. In Taiwan, scholars found that social capital could improve health limitation status [14]. Understanding the positive role social capital plays in health can help policymakers develop more useful policies to promote health.

**Fig. 1:**
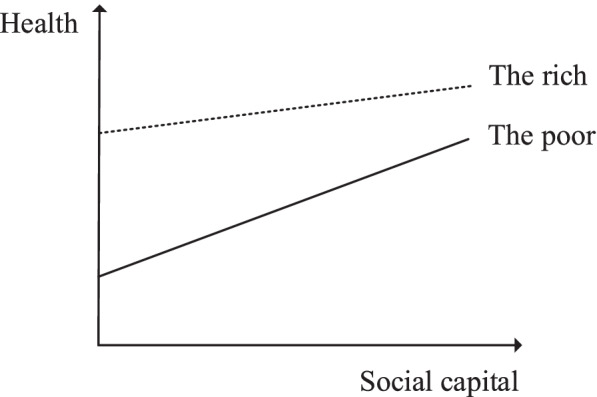
Buffer effect of social capital on health inequality

The effects of social capital on health are reported to vary between different groups, for example, the poor and the rich. The most widely accepted theory is that social capital can provide a buffer effect against the negative influence of some indicators (e.g. poverty) on health [12]. Scholars argue that social capital is, to some degree, the capital to the poor [15, 16]. The cost of time and resources of financial and physical capital of the poor is much lower than the rich; therefore, their health status relies more on social capital [17]. In this way, although social capital can improve both the health condition of the poor and the rich, the benefits to the poor are much more than the rich; thus, social capital can be regarded as a buffer against the negative effect that poverty brings to the poor. Figure 1 exhibits the mechanism that social capital reduces health inequality between the poor and the rich. On the contrary, some scholars hold the opinion that social capital has a dependency effect with income. They argue that sometimes, the poor will be restricted to better use social capital than the rich [18–20]. Consequently, social capital will help the advantaged people benefit more and maintain their privileged position in income level, health status, or other aspects. Zhou concluded that the return of social capital was lower for low-income rural residents than high-income and in this way, social capital became a factor to exacerbate income inequality [21].

So far, what role social capital plays in mental health inequality is still ambiguous. As an important component of individual health conditions, the relationship between mental health inequality and social capital has not been paid enough attention before, especially in China. Mental health, such as depression is gradually more severe in recent years. A national study reported that the prevalence of depressive symptoms was 37.9% and depression 4.1% for Chinese adults [22]. Understanding how social capital affects mental health and how mental health inequality will develop related policies to promote mental health and improve mental health inequality. Therefore, the aim of this study is to explore the relationship between social capital and mental health inequality in China. First, we will analyze the relationship between social capital and mental health. Second, we will conduct an analysis to explore whether social capital has a buffer effect or dependency effect with income level on health, namely, whether social capital will exacerbate or reduce income-related health inequality. Finally, we will analyze the relationship between social capital and mental health inequality in different areas separately, for example, urban and rural areas. In this way, we can better understand how social capital acts in mental health inequality in different institutions and contexts.

## Methods

### Data source

Data is extracted from the China Family Panel Studies (CFPS). CFPS is a nationally representative, longitudinal survey conducted every 2 years since 2010 by Peking University. All individual-, family- and community-level data is collected in CFPS. Information in economic activity, education condition, family relationship, population migration, and physical and mental health is captured in CFPS [23]. The baseline survey was conducted in 2010 and covered 14960 households, 33600 adults, and 8990 children.

Data in 2018 and 2016 of CFPS is used in this current study. 2016 database mainly provides us residents’ social capital information. The lagged variable is widely used to avoid endogeneity [24, 25]. After eliminating those who are under 18, our total sample size contains 25322 adults with 6591 urban respondents and 18731 rural respondents.

## Variables

### Dependent variables

#### Depressive symptoms

The first indicator of mental health is depressive symptoms. Depression is one of the most common mental health disorders in China [26]. Center for Epidemiologic Studies Depression Scale (CES-D) was deployed to measure depressive symptoms in CFPS. CES-D20 was found to be difficult for respondents to finish; thus, in 2018, CES-D20 was replaced by CES-D8. Therefore, in this current study, we used the CES-D8 score to indicate the respondent’s depressive symptoms. In CES-D8, respondents are asked to answer how often in the past week they felt for 8 items, including feeling depressed, feeling everything he did was an effort, sleep restless or not, happy, feeling lonely, enjoying life, feeling sad, and unable to get going. The choices are ranging from ‘none or almost none of the time’ to ‘all or almost or all of the time’, with values 0 to 3. Total scale scores are from 0 to 24, with higher scores representing a higher frequency of depressive symptoms. The validity of CES-D8 has been documented in previous studies [27, 28].

#### Subjective well-being

Another important indicator is well-being. In psychology, the concepts of happiness, well-being, and mental health are often used as synonyms [29]. The World Health Organization defined mental health as a state of well-being in which individuals were able to reach their potential, cope with stresses of life, able to work productively, and make contributions to society [30]. Good mental health indicates an absence of negative symptoms and positive psychological functioning [31]. Hence, it is more and more acknowledged that when measuring mental health, both mental illness (e.g. depression) and well-being should be considered. Mental health is indispensable to subjective well-being, and subjective well-being is the positive side of mental health [31]. Therefore, in this current study, subjective well-being (SWB) is used as another indicator of mental health. Subjective well-being is based on respondents’ answers, instead of pre-judgment of researchers. In CFPS, the respondent was asked “how happy do you think you are”, with values from 0 to 10.

### Independent variables

#### Social capital

In this study, we measured both family-level and community-/village-level social capital. To avoid the endogeneity of social capital and mental health, we used lagged terms in 2016 of these two SCs. In the specific Chinese context, family SC is more related to social networks and guanxi [32, 33]. Guanxi means relation in Chinese. Unlike western society where in-kind gift is common than money gift, in China, money is also widely accepted as a gift, especially on some important occasions such as weddings and New Year Celebration [34]. Giving and receiving money gift has been one of the cores of social relationships in Chinese societies since ancient times [35]. Giving gifts including money and in-kind gifts provides access not only to show filial piety, but also to maintain social networks and acquire social capital [36]. Therefore, we set family gifts as a measurement of family-level social capital. In CFPS, the value of money and in-kind gifts in the past 12 months of all family members given is recorded. The total value of gifts is our indicator of family-level social capital.

As for community-/village-level social capital, neighbor relationship, neighbor assistance, and neighbor feeling are measured. In CFPS 2016, the neighbor relationship was recorded by the question “in general, how do you feel about your neighbor relationship” with five answers from “very good” to “very bad” and we coded them 5, 4, 3, 2, 1, respectively. The neighbor assistance was asked by “if you need help, do you think anyone in your neighborhood will offer assistance”; the answer to this question varied from “there must be” to “there must not be”, with values from 5 to 1. The neighbor sentiment was identified by the question “how much is your feeling to your neighborhood”; the answers were from “very much” to “not at all”, with scale scores from 5 to 1. The Cronbach’s alpha value of these three questions is 0.63, which is regarded as satisfactory and acceptable [37, 38]. Factor analysis of these 3 items is adopted in our study and the result shows a one-factor solution, with eigenvalue equals 1.72 and 57.34% of original information can be explained by factor.

#### Control variables

In our analysis, some variables related to mental health are also included. Income level is indicated by annual household income per capita. This measurement is widely used to analyze income-related health inequality [39]. Scholars recommended that a Body mass index (BMI) of 18.5 to 23.9 should be considered as a standard weight for Chinese people, and 24 to 27.9 as overweight, and >=28 as obese [40, 41]. According to this criterion, we categorize BMI into 4 groups- underweight, normal, overweight, and obese. Education level is divided into 5 groups according to respondent’s highest education qualification, including illiterate or semi-illiterate, junior school and below, high school and technical secondary school, junior college, bachelor and above. Marital status is grouped into unmarried, married, divorced, and widow. Besides, variables including gender, age, health insurance, self-rated health status, currently working or not, rural or urban area, region are all included as control variables.

### Analysis

A series of OLS regression models were conducted to estimate the effects of social capital on mental health and mental health inequality. Firstly, the effects of two kinds of social capital on mental health were examined in total sample, rural sample and urban sample separately. Next, the product term of social capital variables and income level were analyzed to estimate how the effect of SC on mental health varies across different income levels. If the positive effect for the poor is higher than the rich, the mental health disparity can be reduced by social capital (as shown in Fig. 1); otherwise, the disparity will be exacerbated.

In addition, we calculated the marginal effect of social capital on mental health based on the OLS regression results, to analyze whether the effect changes due to the change in income level. The marginal effect of social capital (*s*) on mental health (*y*) is calculated by the following equation:

$$\frac{\partial y}{\partial s}={\beta}_1s+{\beta}_1 Income$$

Where *β*_1_ represents the coefficient of social capital in the OLS model, and *β*_3_ is the coefficient of the interaction term of social capital and income level. In each regression model, we calculated the variance inflation factor (VIF) to analyze the collinearity. The results are shown the Table A1 in the appendix. The VIFs are always less than 10, indicating that there is no collinearity in our models.

## Results

### Descriptive Results

We exhibit the definition and descriptive results of variables included in our analysis in Table 1. From Table 1 we can know that the average CES-D8 score is 5.589, with 4.891 in the urban area and 5.837 in the rural area, respectively. The average SWB score is 7.490, and higher in the urban area. The average value of household gifts is 4496.511 Yuan, with 5450.018 Yuan in urban area and 4160.600 Yuan in the rural area. For community-/village-level social capital, the average scores of neighbor relationship, neighbor assistance, and neighbor feeling in the rural area are all higher than those in the urban area. The average annual household income per capita is 26722.900 Yuan and the income level in the urban area is more than twice the income in the rural area. More than 90% respondents are covered by health insurance. The average age of the total sample is 48.265 years old.

**Table 1:** Descriptive results

Variable	Definition	Total sample		Urban sample		Rural sample	
		Observation	Mean (S.D.)	Observation	Mean (S.D.)	Observation	Mean (S.D.)
**Depressive symptom**	CES-D8 score ranges from 0 to 24	24,996	5.589 (4.031)	6538	4.891 (3.764)	18,458	5.837 (4.092)
**Subjective well-being**	Ranges from 0 to 10	25,145	7.490 (2.157)	6561	7.702 (1.908)	18,584	7.415 (2.234)
**Family-level social capital**	Total values of household money and in-kind gift in the past 12 months	25,158	4496.511 (7734.711)	6554	5450.018 (8489.503)	18,604	4160.600 (7421.734)
**Neighbor relationship**	Ranges from 1 to 5 from *very bad* to *very good*	24,371	2.837 (0.816)	6447	2.795 (0.795)	17,924	2.851 (0.823)
**Neighbor assistance**	Ranges from 1 to 5 from *there must not be* to *there must be*	24,384	3.477 (0.804)	6447	3.407 (0.784)	17,937	3.502 (0.810)
**Neighbor feeling**	Ranges from 1 to 5 from *not at all* to *very much*	24,388	2.946 (0.863)	6450	2.794 (0.826)	17,938	3.001 (0.870)
**Income level**	Annual household income per capita	25,322	26,722.900 (62,339.060)	6587	44,477.140 (74,310.980)	18,731	20,475.600 (56,221.110)
**BMI group**	=0 if underweight; =1 if normal^*^; =2 if overweight; =3 if obese	25,322	1.424 (0.779)	6591	1.487 (0.769)	18,731	1.402 (0.782)
**Education level**	=0 if illiterate or semi-illiterate^*^; =1 if junior school and below; =2 if high school and technical secondary school; =3 if junior college; =4 if bachelor and above	25,322	1.211 (1.035)	6591	1.834 (1.189)	18,731	0.992 (0.876)
**Health insurance**	=0 if not join any health insurance^*^; =1 if join any health insurance	24,932	0.922 (0.268)	6503	0.904 (0.295)	18,429	0.928 (0.258)
**Self-rated health status**	Ranges from 0 to 4 from *very bad* to *very good*	25,080	1.931 (1.219)	6555	1.906 (1.093)	18,525	1.940 (1.261)
**Working status**	=0 if not working^*^; =1 if currently working	24,437	0.705 (0.456)	6366	0.581 (0.494)	18,071	0.749 (0.434)
**Marital status**	=0 if unmarried^*^; =1 if married; =2 if divorce; =3 if widow	25,322	1.023 (0.610)	6591	1.034 (0.618)	18,731	1.019 (0.607)
**Rural**	=0 if in urban area^*^ =1 if in rural area	25,322	0.740 (0.439)				
**Gender**	=0 if female^*^; =1 if male	25,322	0.496 (0.500)	6591	0.509 (0.500)	18,731	0.492 (0.500)
**Age**		25,322	48.260 (16.276)	6591	49.785 (16.583)	18,731	47.723 (16.132)

Note: * reference group for all regressions

**Fig. 2:**
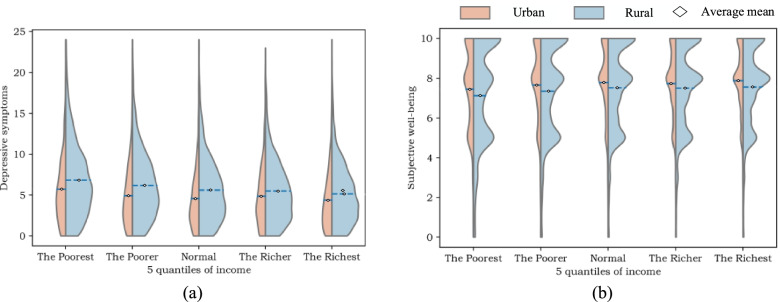
Distribution of mental health indicators

Figure 2 exhibits the distribution of depressive symptoms and SWB among people with different income levels. We can learn from Fig. 2 that both in urban and rural areas, people with higher income level possess lower level of depressive symptoms, while in terms of subjective well-being, no significant decline of SWB has been observed as income level increases.

**Fig. 3:**
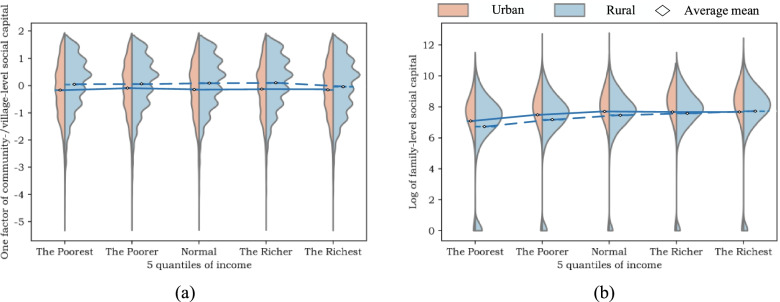
Distribution of mental health indicators

Figure 3 displays the distribution of family-level SC and community-/village-level SC among people with different income levels. It can be concluded that as income level grows, the amount of family-level SC increases, and the urban residents possess more family-level SC than the rural residents, while the rural residents possess more community-/village-level SC than the urban residents, and no significant increase of community-/village-level SC has been observed as income level increases.

### OLS Regression

#### Depression Symptoms

The effect of social capital and income level on depressive symptoms is shown in model 1 in Table 2. In model 2 and model 3, we added the product term of two kinds of social capital and income level. From model 1, we can conclude that social capitals, including family-level SC and community-/village-level SC have a significant effect to inhibit depressive symptoms in the population. The coefficient of income level indicates that people with higher income are less likely to be depressed, which suggests that mental health is severely unequal among Chinese residents. People overweight or obese are less likely to have more depressive symptoms, while people who are underweight have higher depressive symptoms compared with normal-weight people. Additionally, higher education level, joining health insurance, higher self-rated health status, being married, male, living in middle or eastern region and elderly people are all showed less likely to be depressed, while working and being divorced or widowed are positively related to depressive symptom. The coefficient of interaction terms in model 2 and model 3 suggests that the effect of social capital doesn’t diverse among people with different income levels in the total sample. That is to say, the health return of social capital of the rich is the same as the poor. Consequently, social capital doesn’t play any role in mental health inequality.

**Table 2:** Social capital and depressive symptom in the total sample

	Model 1	Model 2	Model 3
Family-level SC	−0.026^**^ (0.013)	−0.191^*^ (0.113)	−0.026^**^ (0.013)
Community−/Village- level SC	− 0.497^***^ (0.028)	− 0.497^***^ (0.028)	− 0.753^***^ (0.273)
Log of income level	− 0.370^***^ (0.034)	− 0.491^***^ (0.089)	− 0.369^***^ (0.034)
Family-level SC × log of income level		0.017 (0.011)	
Community−/Village- level SC × log of income level			0.027 (0.028)
BMI group			
Underweight	0.383^***^ (0.108)	0.381^***^ (0.108)	0.382^***^ (0.108)
Overweight	−0.179^***^ (0.055)	− 0.179^***^ (0.055)	− 0.179^***^ (0.055)
Obese	− 0.265^***^ (0.089)	− 0.266^***^ (0.089)	− 0.265^***^ (0.089)
Education level			
Junior school and below	−0.601^***^ (0.075)	− 0.601^***^ (0.075)	− 0.602^***^ (0.075)
High school and technical secondary school	−0.947^***^ (0.095)	− 0.950^***^ (0.095)	− 0.947^***^ (0.095)
Junior college	− 0.855^***^ (0.122)	− 0.862^***^ (0.122)	− 0.854^***^ (0.122)
Bachelor and above	− 0.853^***^ (0.132)	− 0.865^***^ (0.132)	− 0.848^***^ (0.132)
Health insurance	− 0.601^***^ (0.104)	− 0.602^***^ (0.104)	− 0.601^***^ (0.104)
Self-rated health status	−1.013^***^ (0.023)	−1.013^***^ (0.023)	−1.013^***^ (0.023)
Working	0.125^**^ (0.062)	0.126^**^ (0.062)	0.125^**^ (0.062)
Marital status			
Married	−0.301^***^ (0.103)	−0.301^***^ (0.103)	− 0.305^***^ (0.104)
Divorced	1.151^***^ (0.219)	1.152^***^ (0.218)	1.147^***^ (0.219)
Widowed	1.470^***^ (0.177)	1.468^***^ (0.177)	1.466^***^ (0.177)
Rural	0.391^***^ (0.067)	0.391^***^ (0.067)	0.392^***^ (0.067)
Male	−0.540^***^ (0.048)	−0.540^***^ (0.048)	− 0.539^***^ (0.049)
Region			
Middle	−0.432^***^ (0.074)	−0.434^***^ (0.074)	− 0.432^***^ (0.074)
Eastern	−0.632^***^ (0.069)	− 0.635^***^ (0.069)	− 0.630^***^ (0.069)
Age	− 0.021^***^ (0.002)	− 0.022^***^ (0.002)	− 0.021^***^ (0.002)
R^2^	0.1821	0.1822	0.1821
N	22,969	22,969	22,969

Note: *,**,***: significantly different from zero at the 0.1, 0.05 and 0.01 level, respectively; standard errors are clustered by family

Due to different living customs and economic developments in urban and rural areas, we conducted the above models in urban and rural samples separately, which is shown in Table 3. The regression results in model 4 and model 7 indicate that education, health insurance, self-rated health scores, middle and eastern areas, age are all positively related to lower CES-D8 scores in both urban and rural areas. Underweight people are likely to have more severe depressive symptoms than normal people in the rural area, and obese population are less likely to be depressed in the urban area. Both family SC and community/village SC shows a negative effect on CES-D8 scores in the urban area. However, in rural area, the coefficient of family SC is not statistically significant. Not surprisingly, income level has a negative relationship with CES-D8, which means that the rich are less likely to be depressed than the poor. The results in model 4 and 7 preliminarily validate that there exists income-related inequality in depression, and social capital provides effective access to prevent depression.

**Table 3:** Social capital and depressive symptom in urban and rural samples

	Urban area			Rural area		
	Model 4	Model 5	Model 6	Model 7	Model 8	Model 9
Family-level SC	−0.066^***^ (0.022)	− 0.672^***^ (0.255)	− 0.066^***^ (0.022)	−0.008 (0.015)	− 0.170 (0.145)	−0.008 (0.015)
Community−/Village- level SC	−0.472^***^ (0.051)	− 0.472^***^ (0.051)	−1.216^*^ (0.633)	− 0.510^***^ (0.033)	− 0.510^***^ (0.033)	− 0.671^**^ (0.326)
Log of income level	− 0.400^***^ (0.068)	− 0.812^***^ (0.191)	− 0.392^***^ (0.068)	− 0.332^***^ (0.039)	− 0.457^***^ (0.117)	− 0.332^***^ (0.038)
Family-level SC × log of income level		0.058^**^ (0.024)			0.017 (0.015)	
Community−/Village- level SC × log of income level			0.072 (0.061)			0.017 (0.034)
BMI group						
Underweight	0.245 (0.218)	0.226 (0.219)	0.236 (0.219)	0.412^***^ (0.124)	0.412^***^ (0.124)	0.412^***^ (0.124)
Overweight	−0.217^**^ (0.099)	−0.218^**^ (0.099)	−0.216^**^ (0.099)	− 0.171^***^ (0.065)	−0.171^***^ (0.065)	− 0.171^***^ (0.065)
Obese	−0.544^***^ (0.153)	− 0.549^***^ (0.153)	− 0.541^***^ (0.153)	− 0.175 (0.108)	−0.176 (0.108)	− 0.175 (0.108)
Education level						
Junior school and below	−0.457^**^ (0.202)	− 0.460^**^ (0.203)	− 0.459^**^ (0.202)	− 0.592^***^ (0.082)	− 0.593^***^ (0.082)	−0.593^***^ (0.082)
High school and technical secondary school	−0.913^***^ (0.212)	− 0.926^***^ (0.212)	− 0.917^***^ (0.212)	−0.867^***^ (0.113)	− 0.870^***^ (0.113)	−0.867^***^ (0.113)
Junior college	− 0.646^***^ (0.238)	0.660^***^ (0.239)	− 0.648^***^ (0.238)	−1.044^***^ (0.158)	− 1.050^***^ (0.158)	− 1.044^***^ (0.158)
Bachelor and above	− 0.634^**^ (0.248)	− 0.663^***^ (0.248)	− 0.628^**^ (0.248)	− 1.386^***^ (0.187)	− 1.396^***^ (0.188)	− 1.382^***^ (0.187)
Health insurance	− 0.721^***^ (0.184)	− 0.705^***^ (0.184)	− 0.719^***^ (0.184)	− 0.521^***^ (0.126)	− 0.521^***^ (0.126)	− 0.521^***^ (0.126)
Self-rated health status	− 1.040^***^ (0.046)	− 1.037^***^ (0.046)	− 1.040^***^ (0.046)	−1.001^***^ (0.026)	− 1.002^***^ (0.026)	− 1.001^***^ (0.026)
Working	0.370^***^ (0.114)	0.367^***^ (0.114)	0.371^***^ (0.114)	−0.049 (0.075)	− 0.047 (0.075)	− 0.049 (0.075)
Marital status						
Married	−0.266 (0.210)	− 0.268 (0.209)	− 0.271 (0.210)	− 0.393^***^ (0.120)	− 0.393^***^ (0.120)	− 0.396^***^ (0.120)
Divorced	0.804^**^ (0.343)	0.808^**^ (0.342)	0.796^**^ (0.343)	1.308^***^ (0.029)	1.306^***^ (0.289)	1.306^***^ (0.289)
Widowed	1.598^***^ (0.339)	1.582^***^ (0.338)	1.594^***^ (0.339)	1.318^***^ (0.208)	1.317^***^ (0.208)	1.315^***^ (0.208)
Male	−0.592^***^ (0.090)	− 0.588^***^ (0.090)	− 0.589^***^ (0.090)	−0.519^***^ (0.058)	− 0.519^***^ (0.058)	− 0.519^***^ (0.058)
Region						
Middle	−0.289^**^ (0.145)	−0.288^**^ (0.145)	− 0.286^**^ (0.145)	− 0.506^***^ (0.085)	− 0.507^***^ (0.085)	− 0.506^***^ (0.085)
Eastern	−0.615^***^ (0.138)	− 0.614^***^ (0.138)	− 0.608^***^ (0.138)	− 0.627^***^ (0.079)	− 0.630^***^ (0.079)	− 0.626^***^ (0.079)
Age	−0.030^***^ (0.005)	− 0.030^***^ (0.005)	− 0.030^***^ (0.005)	− 0.017^***^ (0.003)	− 0.017^***^ (0.003)	− 0.017^***^ (0.003)
R^2^	0.1677	0.1689	0.1680	0.1774	0.1775	0.1774
N	6099	6099	6099	16,870	16,870	16,870

Note: *,**,***: significantly different from zero at the 0.1, 0.05 and 0.01 level, respectively; standard errors are clustered by family

**Fig. 4:**
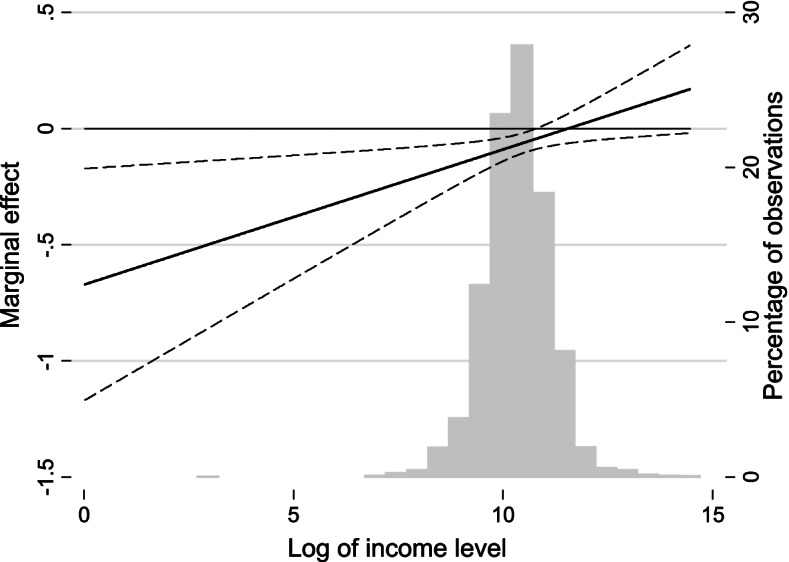
Marginal effect of family-level SC on depressive symptoms in the urban area

The coefficient of the interaction term in model 5 indicates that the inhibiting effect of SC on depression is pro-poor. In other words, the negative effect of SC on depression diminishes as income level increases. We already know from model 4 that family-level SC produces a positive return on mental health among urban residents. We can furtherly conclude from model 5 that for the poor, the health return of one unit of family-level SC is much higher than the rich. The marginal effect of family-level SC on CES-D8 is shown in Fig. 4. This figure can better explain the relationship between SC, income level, and CES-D8. The grey column represents the percentage of observations located in total scales of income level. The solid line indicates marginal effect and dotted lines mark the 95% confidential intervals. We can learn from Fig. 4 that the marginal effect is negative however gradually weaker as income increases. Hence, we can conclude that with the effect of family-level SC, the mental health inequality in the urban area will be weakened than before. The coefficients of interaction terms in model 6, model 8, and model 9 are also positive, however, not statistically significant.

#### Subjective well-being

Table 4 exhibits our regression results of subjective well-being in the total sample. The dependent variable in these three models is the SWB of respondents. Independent variables in model 1, model 2, and model 3 are consistent with models 1, 2, and 3 in Table 2. The results in model 1 suggest family-level SC, community-/village-level SC, income level, BMI, health insurance, self-rated health status, and age are all positively related to the respondent’s SWB. In the meanwhile, overweight and obese populations are proved to have a higher level of SWB than the normal weight population; people whose highest education level are junior college or bachelor and above are more likely to have higher SWB; residents living in eastern or middle China have better SWB than those living in western China. However, people who are currently working, divorced, living in rural areas, or male are estimated to have lower SWB. In model 2 and model 3, we added the interaction term of SC and log of income level in our regression. The 5^th^ line in model 3 indicates that the return of community-/village-level SC on health is pro-poor, which proves the buffer effect of social capital on mental health inequality.

**Table 4:** Social capital and subjective well-being in the total sample

	Model 1	Model 2	Model 3
Family-level SC	0.020^***^ (0.007)	0.072 (0.063)	0.020^***^ (0.007)
Community−/Village- level SC	0.339^***^ (0.016)	0.339^***^ (0.016)	0.608^***^ (0.157)
Log of income level	0.121^***^ (0.019)	0.159^***^ (0.049)	0.120^***^ (0.019)
Family-level SC × log of income level		−0.005 (0.006)	
Community−/Village- level SC × log of income level			−0.028^*^ (0.016)
BMI group			
Underweight	0.095 (0.058)	0.096^*^ (0.058)	0.096^*^ (0.058)
Overweight	0.053^*^ (0.031)	0.054^*^ (0.031)	0.054^*^ (0.031)
Obese	0.224^***^ (0.048)	0.225^***^ (0.048)	0.225^***^ (0.048)
Education level			
Junior school and below	0.024 (0.042)	0.024 (0.042)	0.024 (0.042)
High school and technical secondary school	0.044 (0.054)	0.045 (0.054)	0.045 (0.054)
Junior college	0.202^***^ (0.067)	0.204^***^ (0.067)	0.201^***^ (0.067)
Bachelor and above	0.249^***^ (0.070)	0.253^***^ (0.071)	0.244^***^ (0.071)
Health insurance	0.130^**^ (0.060)	0.131^**^ (0.060)	0.130^**^ (0.060)
Self-rated health status	0.356^***^ (0.013)	0.356^***^ (0.013)	0.356^***^ (0.013)
Working	−0.186^***^ (0.035)	−0.186^***^ (0.035)	−0.185^***^ (0.035)
Marital status			
Married	0.327^***^ (0.060)	0.327^***^ (0.060)	0.332^***^ (0.060)
Divorced	−0.838^***^ (0.125)	− 0.838^***^ (0.125)	− 0.833^***^ (0.125)
Widowed	−0.107 (0.097)	− 0.107 (0.097)	− 0.103 (0.097)
Rural	− 0.119^***^ (0.037)	− 0.119^***^ (0.037)	− 0.119^***^ (0.037)
Male	− 0.080^***^ (0.027)	− 0.080^***^ (0.027)	− 0.081^***^ (0.027)
Region			
Middle	0.306^***^ (0.041)	0.307^***^ (0.041)	0.306^***^ (0.041)
Eastern	0.326^***^ (0.039)	0.327^***^ (0.039)	0.324^***^ (0.039)
Age	0.011^***^ (0.001)	0.011^***^ (0.001)	0.011^***^ (0.001)
R^2^	0.0963	0.0963	0.0965
N	23,035	23,035	23,035

Note: *,**,***: significantly different from zero at the 0.1, 0.05 and 0.01 level, respectively; standard errors are clustered by family

We also conducted the same regressions in urban and rural areas separately. The results are shown in Table 5. Model 4 and model 7 indicate that higher levels of social capital and income are related to a higher level of SWB. Different from the rural sample, people in the urban area who are underweight or overweight likely to be happier, while higher education levels are proved to have no positive impact on SWB. Health insurance can positively affect SWB in the urban area rather than the rural area. The coefficients of interaction terms in model 5, model 6, and model 8 are neither statistically significant, while that in model 9 is negative and statistically significant. These results show that both SCs don’t play any role in the inequality of SWB in the urban area; however, in the rural area, the positive effect of village-level SC on SWB decreases as income level increases.

**Table 5:** Social capital and subjective well-being in urban and rural samples

	Urban area			Rural area		
	Model 4	Model 5	Model 6	Model 7	Model 8	Model 9
Family-level SC	0.030^***^ (0.011)	0.171 (0.134)	0.030^***^ (0.012)	0.015^*^ (0.009)	0.077 (0.080)	0.015^*^ (0.009)
Community−/Village- level SC	0.320^***^ (0.027)	0.320^***^ (0.027)	0.232 (0.347)	0.347^***^ (0.019)	0.347^***^ (0.019)	0.686^***^ (0.189)
Log of income level	0.147^***^ (0.035)	0.243^**^ (0.100)	0.148^***^ (0.035)	0.106^***^ (0.022)	0.154^**^ (0.064)	0.105^***^ (0.022)
Family-level SC × log of income level		−0.014 (0.013)			−0.007 (0.008)	
Community−/Village- level SC × log of income level			0.009 (0.033)			−0.036^*^ (0.019)
BMI group						
Underweight	0.196^*^ (0.105)	0.201^*^ (0.105)	0.195^*^ (0.105)	0.073 (0.068)	0.073 (0.068)	0.073 (0.068)
Overweight	0.117^**^ (0.051)	0.117^**^ (0.051)	0.117^**^ (0.051)	0.027 (0.038)	0.027 (0.038)	0.028 (0.038)
Obese	0.157^*^ (0.082)	0.158^*^ (0.082)	0.157^*^ (0.082)	0.251^***^ (0.058)	0.252^***^ (0.058)	0.252^***^ (0.058)
Education level						
Junior school and below	−0.037 (0.104)	−0.036 (0.104)	− 0.037 (0.104)	0.027 (0.047)	0.027 (0.047)	0.028 (0.047)
High school and technical secondary school	−0.085 (0.109)	−0.082 (0.109)	− 0.086 (0.110)	0.091 (0.065)	0.092 (0.065)	0.091 (0.065)
Junior college	0.148 (0.121)	0.151 (0.121)	0.148 (0.121)	0.196^**^ (0.089)	0.198^**^ (0.089)	0.195^**^ (0.089)
Bachelor and above	0.120 (0.125)	0.126 (0.126)	0.120 (0.125)	0.423^***^ (0.102)	0.427^***^ (0.102)	0.413^***^ (0.102)
Health insurance	0.190^*^ (0.099)	0.186^*^ (0.099)	0.190^*^ (0.099)	0.097 (0.076)	0.097 (0.077)	0.098 (0.076)
Self-rated health status	0.318^***^ (0.024)	0.318^***^ (0.024)	0.319^***^ (0.024)	0.365^***^ (0.015)	0.365^***^ (0.015)	0.365^***^ (0.015)
Working	−0.324^***^ (0.061)	−0.323^***^ (0.061)	− 0.324^***^ (0.061)	− 0.111^**^ (0.043)	−0.112^***^ (0.043)	− 0.111^**^ (0.043)
Marital status						
Married	0.249^**^ (0.101)	0.249^**^ (0.101)	0.248^**^ (0.101)	0.376^***^ (0.074)	0.376^***^ (0.074)	0.383^***^ (0.074)
Divorced	−0.742^***^ (0.178)	−0.743^***^ (0.178)	− 0.743^***^ (0.178)	− 0.896^***^ (0.171)	− 0.895^***^ (0.171)	− 0.892^***^ (0.170)
Widowed	− 0.237 (0.166)	− 0.234 (0.166)	− 0.238 (0.167)	−0.027 (0.119)	− 0.027 (0.119)	−0.021 (0.119)
Male	0.020 (0.047)	0.019 (0.047)	0.020 (0.047)	−0.118^***^ (0.034)	−0.118^***^ (0.034)	− 0.119^***^ (0.034)
Region						
Middle	0.256^***^ (0.078)	0.256^***^ (0.078)	0.256^***^ (0.078)	0.323^***^ (0.047)	0.323^***^ (0.047)	0.322^***^ (0.047)
Eastern	0.243^***^ (0.074)	0.243^***^ (0.074)	0.244^***^ (0.074)	0.346^***^ (0.045)	0.347^***^ (0.045)	0.344^***^ (0.045)
Age	0.011^***^ (0.002)	0.012^***^ (0.002)	0.011^***^ (0.002)	0.010^***^ (0.002)	0.010^***^ (0.002)	0.010^***^ (0.002)
R^2^	0.1020	0.1022	0.1020	0.0925	0.0925	0.0927
N	6113	6113	6113	16,922	16,922	16,922

Note: *,**,***: significantly different from zero at the 0.1, 0.05 and 0.01 level, respectively; standard errors are clustered by family

**Fig. 5:**
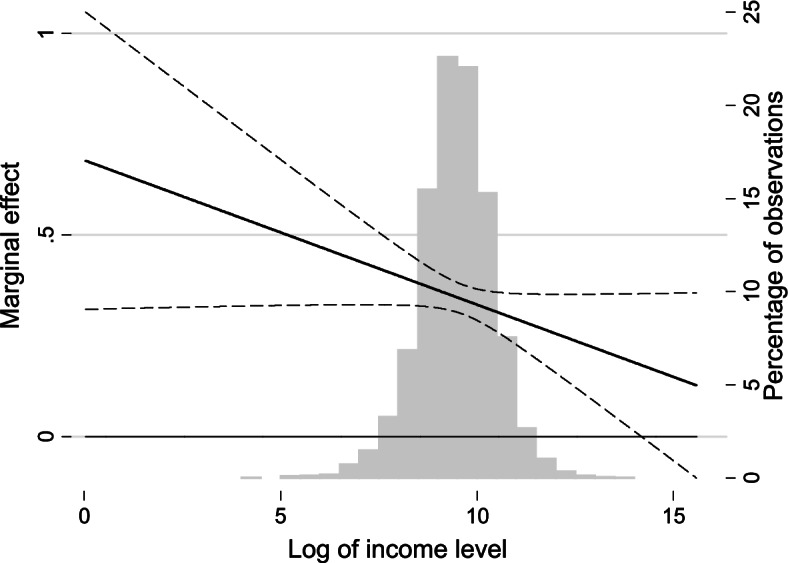
Marginal effect of community-/village-level SC on subjective well-being in the rural area

The marginal effect in Fig. 5 can help us understand the role village-level SC plays in the income-related inequality of SWB. The marginal effect is positive which suggests that village-level SC has a positive return to SWB. As the income level increases, the marginal effect diminishes and draws near to 0, and finally, the marginal effect of village-level SC becomes not statistically significant. From the results in model 9 and Fig. 5, we can conclude that village SC can narrow the SWB disparity between the poor and the rich in the rural area.

### Subsample analysis

Considering that family-level SC guanxi could have different impacts on mental health and corresponding inequality, we extract those who are the financial head of households. The CFPS didn’t survey whether the respondent was head of household [42]. The survey team recommended that scholars could use other variables for different contexts and situations (http://www.isss.pku.edu.cn/cfps/cjwt/cfpsxkt/1323217.htm). We set the financial head of household as the head of family [43], which was obtained by the survey question “*Who is the family member who is most familiar with household income and expenditure over the past 12 months?*”. Totally, 11562 respondents are financial heads of households and among them, 6046 are male. We re-analyzed the relation between family-level SC, mental health and mental health inequality. The results are presented in Table 6.

**Table 6:** Family-level social capital and mental health among household financial head sample

	Depressive symptoms						Subjective well-being					
	Total		Urban area		Rural area		Total		Urban area		Rural area	
	Model 1	Model 2	Model 3	Model 4	Model 5	Model 6	Model 7	Model 8	Model 9	Model 10	Model 11	Model 12
Family-level SC	−0.025 (0.016)	−0.199 (0.128)	− 0.042 (0.027)	− 0.580^**^ (0.269)	− 0.018 (0.020)	− 0.104 (0.164)	0.013 (0.009)	0.089 (0.070)	0.013 (0.014)	0.181 (0.137)	0.012 (0.011)	0.072 (0.092)
Community−/Village- level SC	−0.513^***^ (0.037)	−0.513^***^ (0.037)	− 0.447^***^ (0.068)	−0.448^***^ (0.068)	− 0.295^***^ (0.044)	−0.546^***^ (0.044)	0.345^***^ (0.020)	0.345^***^ (0.020)	0.329^***^ (0.035)	0.329^***^ (0.035)	0.355^***^ (0.025)	0.355^***^ (0.025)
Log of income level	−0.380^***^ (0.042)	−0.504^***^ (0.100)	− 0.525^***^ (0.088)	−0.877^***^ (0.196)	− 0.547^***^ (0.049)	− 0.361^***^ (0.133)	0.160^***^ (0.023)	0.215^***^ (0.055)	0.201^***^ (0.045)	0.311^***^ (0.010)	0.137^***^ (0.028)	0.182^**^ (0.075)
Family-level SC × log of income level		0.018 (0.013)		0.052^**^ (0.068)		0.009 (0.017)		− 0.008 (0.007)		− 0.016 (0.013)		− 0.006 (0.010)
Control variables	Yes	Yes	Yes	Yes	Yes	Yes	Yes	Yes	Yes	Yes	Yes	Yes
R^2^	0.1986	0.1986	0.1876	0.1884	0.1945	0.1945	0.1072	0.1073	0.1235	0.1236	0.0983	0.0982
Observations	11,012	11,012	3060	3060	7952	7952	11,050	11,050	3067	3067	7983	7983

Note: *,**,***: significantly different from zero at the 0.1, 0.05 and 0.01 level, respectively; control variables in total sample include BMI group, education level, health insurance, self-rated health status, working, marital status, rural, male, region and age; control variables in urban/rural samples include BMI group, education level, health insurance, self-rated health status, working, marital status, male, region and age

From the results of model 1, model 3, model 5, model 7, model 9 and model 11 in Table 6, we can conclude that family-level SC doesn’t have any observed positive impact on family heads’ mental health, neither measured by depressive symptoms, nor by SWB. The coefficient of the interaction term in model 4 in Table 6 indicates that the family-level SC can buffer mental health inequality, which shows the same result with model 5 in Table 3.

## Discussion

Does social capital worsen or narrow mental health inequality? Is the mental health gap between the poor and the rich enlarged or closed by social capital? In this study, we measured both family-level and community-/village-level social capitals and investigated their associations with mental health and income-related mental health inequality. The results of this current study contribute to relevant literature on how to promote mental health and decrease mental health inequality.

Firstly, our results validate the positive effect of social capital on mental health. Our results indicate that a higher level of community/village SC is related to fewer depressive symptoms in both urban and rural areas, and family SC also has a negative impact on depressive symptoms in the urban area. These findings are consistent with previous research on social capital and depression. An existing study focusing on the effect of social capital on depression among Chinese urban elderly suggests that trust, reciprocity, and social network are all significantly associated with lower geriatric depression [44]. A study conducted in other countries also provides similar results [45]. For SWB, both family-level social capital and community-/village- level social capital play a positive role no matter in the urban or rural areas. This finding is consistent with some previous research. Several studies have reported that social capital originated from social networks and neighborhood relationships could improve residents’ happiness [46, 47]. China is a typical relational society. Scholars stated that the behaviors of giving and receiving gifts were based on the principle of reciprocity [48]. A Chinese scholar furtherly pointed out that giving and receiving gifts in Chinese society could help to preserve the partnership among intimate associations [49]. Guanxi or relation plays an essential role in socio-economic development, and is regarded to be an important way to gain social capital. In this study, we set the total value of money and in-kind gifts in the past 12 months as family-level guanxi. This approach has been adopted by other scholars when measuring social capital in the Chinese context [50]. However, when measuring individual’s SWB, money/in-kind gift as social capital hasn’t been considered while it’s very important to mental health. Our results contribute to the existing literature on the measurement of social capital and improvement of SWB.

Unlike village-level SC, family-level SC, measured by household gift in this study, is found to be unrelated to depressive symptoms in the rural area. Other scholars also have found that community-/village-level SC has a much greater effect on depression This may be possibly due to the intimate correlation within village. In rural China, a village was usually originated from a clan or tribe. Villagers have tighter relations and thus, they rely less on the relationship within family compared with urbanites [51]. Another potential reason is that in rural China, the function of giving gifts is to keep relative standing and cope with competitive mating pressure rather than maintaining social network and social capital [52]. The subsample analysis among household financial heads indicates that family-level social capital doesn’t have an observed impact on mental health. This may be because the financial head is the person who is most familiar with the household’s income and expenditure. Although social capital could bring mental health benefits to residents, it also needs economic costs for maintaining social capital. Therefore, the one who manages household’s property needs to bear the costs of giving money or in-kind gifts, which reduces the benefits brought by social capital. Previous studies have also documented that household heads experienced more mental stress due to household food burden and financial expenditure [53, 54].

Secondly, in terms of social capital and mental health inequality, this current study provides interesting findings. Although whether social capital brings buffer effect or dependency effect on health inequality has been a commonplace topic and scholars found different answers for this question, our regression results and decomposition results prove that social capital has a buffer effect on mental health inequality. For the urban residents, family-level SC has a greater effect on inhibiting depressive symptoms for the poor, and for the rural residents, village-level SC has a greater effect on improving SWB for the poor. These findings confirm the idea that *social capital is the capital of the poor*. Some scholars have been arguing that social capital could improve health but would also exacerbate health inequalities, because not everyone would benefit in the same way. People with a low-income level usually have less social capital; moreover, they have limited social capital to make full use of to promote health. However, the buffer effect indicates that social capital has a greater effect on health for the disadvantaged, and no effect or limited health benefits for the advantaged. A previous study conducted in America concluded that especially for American Jewish with low income, religious bonding can significantly improve self-rated health [55]. Another study conducted in Latin American also found that generalized and neighborhood trust could modify the effect of SES on health, especially for low SES groups [56]. Researches focusing on China also found similar results. Research conducted in rural China concluded that social networks could improve the well-being of rural residents, and the positive effect was much more significant for low-income groups [16]. Similarly, a study conducted in China showed that only for the poor, social capital was statistically associated with self-rated health [57]. There are several potential reasons for our results. The first potential explanation is that the poor possess much more time to manage their social network than the rich. Usually, the rich don’t have as much leisure time as the poor have, so the poor can make use of their leisure time to benefit from social participation and networks. Another potential reason is as Collier noted that the poor had a smaller amount of physical capital or economic capital; thus, they had to rely more on social capital as a substitution of physical or economic capitals [17].

Thirdly, we find that mental health in urban area is much better than in the rural area. This is consistent with previous findings. Our results also suggest that income level is strongly associated with mental health, both depressive symptoms and subjective well-being. Plenty of previous literature suggests that income-related mental health inequality exists all around the world [58–60]. Additionally, education level, health insurance, being married, and self-rated health status are positively associated with mental health. These findings are consistent with previous findings [61]. However, the regression results show some differences. We find that people with higher BMI have higher mental health status, while in another research, BMI is negatively related to better mental health [62]. This unusual result has also been reported in several other literatures [63–65]. With more and more research finding that overweight or obese patients usually live longer, scholars concluded the existence of so-called “obesity paradox”. Our results are consistent with the findings of the “obesity paradox”. In addition, while other researchers found that employed people usually had better mental health [66], our finding reports that work could decrease personal mental health. A potential explanation for this result is that the survey in CFPS measured working status but not employment status. Currently working means less leisure time and more physical burden, thus brings poorer mental health. However, previous literature usually measures the effect of being employed or unemployed on mental health. Being employed implies a stable income and work environment.

It should be noted that reducing poverty is still the most important way to improve the mental health status of the poor. However, our findings on the relationship between social capital and mental health inequality provide some new ideas to improve the poor’s mental health status and reduce mental health inequality. For example, we advocate establishing more vulnerable groups in the community or village, especially for the disadvantaged people to expand their social network. Measures should be taken to promote social participation of the poor, such as recommending them to join social organizations. In the rural area, the service quality of the village committee and the participation of villagers should be enhanced to improve their village-level social capital, especially for people with a low-income level.

We want to caution against several limitations in this study. The first limitation is that the data we used was cross-sectional and hence we couldn’t provide a causal analysis. However, we used the lagged terms of social capital to avoid the endogeneity of social capital and mental health. This is the best solution to our problem because we tried to find a perfect instrumental variable but failed. The second limitation is the self-reported happiness indicator we used. Because different people have different definitions of happiness, subjective well-being may cause some bias to measure the happiness status of residents. Despite these limitations, our study still provides important evidence on the relationship between social capital, mental health, and mental health inequality in China.

## Conclusion

This study investigates whether social capital buffers or exacerbates mental health inequality. The results show that severe mental health inequality exists in China; family-level social capital can buffer depressive symptom inequality, and village-level social capital can buffer SWB inequality. Our study advocate enhancing social participation and communication of the poor.

## Supplementary Information

**Additional file 1.**

## Data Availability

The datasets generated and analyzed during the current study were derived from the China Family Panel Study (CFPS). They are opened to everyone. Researchers who want to use these data can visit www.isss.pku.edu.cn/cfps/.

## References

[1] Fang P, Dong S, Xiao J, Liu C, Feng X, Wang Y (2010). Regional inequality in health and its determinants: evidence from China. Health Policy Plan.

[2] Fan C, Ouyang W, Tian L, Song Y, Miao W (2019). Elderly health inequality in China and its determinants: a geographical perspective. Int J Environ Res Public Health.

[3] Zhou Z, Fang Y, Zhou Z, Li D, Wang D, Li Y, Lu L, Gao J, Chen G (2017). Assessing income-related health inequality and horizontal inequity in China. Soc Indicat Res.

[4] Wang H, Yu Y (2016). Increasing health inequality in China: an empirical study with ordinal data. Jf Econ Inequal.

[5] Strauss J, Lei X, Park A, Shen Y, Smith JP, Yang Z, Zhao Y (2010). Health outcomes and socio-economic status among the elderly in China: Evidence from the CHARLS Pilot. J Pop Ageing.

[6] Lei X, Sun X, Strauss J, Zhang P, Zhao Y (2014). Depressive symptoms and SES among the mid-aged and elderly in China: Evidence from the China Health and Retirement Longitudinal Study national baseline. Soc Sci Med.

[7] Guo J, Guan L, Fang L, Liu C, Fu M, He H, Wang X (2017). Depression among Chinese older adults: A perspective from Hukou and health inequities. J Affect Disord.

[8] Bourdieu P (2008). The Forms of Capital: Readings in Economic Sociology.

[9] Coleman, James S: Frontmatter: Grundlagen der Sozialtheorie [Foundations of Social Theory]. Handlungen und Handlungssysteme 1991.

[10] Putnam RD, Leonardi DR (1994). Making Democracy Work: Civic Traditions in Modern Italy. Contemp Sociol.

[11] Yip W, Subramanian SV, Mitchell AD, Lee DT, Wang J, Kawachi I (2007). Does social capital enhance health and well-being? Evidence from rural China. Soc Sci Med.

[12] Uphoff EP, Pickett KE, Cabieses B, Small N, Wright J (2013). A systematic review of the relationships between social capital and socioeconomic inequalities in health: a contribution to understanding the psychosocial pathway of health inequalities. Int J Equity Health.

[13] Liu GG, Xue X, Yu C, Wang Y (2016). How does social capital matter to the health status of older adults? Evidence from the China Health and Retirement Longitudinal Survey. Econ Hum Biol.

[14] Song L, Pettis PJ (2020). Does whom you know in the status hierarchy prevent or trigger health limitation? Institutional embeddedness of social capital and social cost theories in three societies. Soc Sci Med.

[15] Zhou G, Fan G, Shen G: The Income Disparity, the Social Capital and Health: A Case Study Based on China Family Panel Studies. J Manag World (in Chinese) 2014, 000(007):12-21,51.

[16] Li S, Chen G (2012). Does guanxi brings happiness? Evidence from rural China. Chin Rural Econ (in Chinese).

[17] Collier P (2002). The Role of Social Capital in Development: Social capital and poverty: a microeconomic perspective.

[18] Baron-Epel O, Weinstein R, Haviv-Mesika A, Garty-Sandalon N, Green MS (2008). Individual-level analysis of social capital and health: a comparison of Arab and Jewish Israelis. Soc Sci Med.

[19] Beaudoin CE (2009). Social capital and health status: assessing whether the relationship varies between Blacks and Whites. Psychol Health.

[20] Gorman BK, Sivaganesan A (2007). The role of social support and integration for understanding socioeconomic disparities in self-rated health and hypertension. Soc Sci Med.

[21] Zhou Y (2012). Is social capital the capital of the poor? Evidence nased on income of rural residents in China. J Manag World (in Chinese).

[22] Qin X, Wang S, Hsieh C-R (2018). The prevalence of depression and depressive symptoms among adults in China: estimation based on a National Household Survey. China Econ Rev.

[23] Xie Y, Hu J (2014). An introduction to the China family panel studies (CFPS). Chin Sociol Rev.

[24] Clemens MA, Radelet S, Bhavnani RR, Bazzi S (2012). Counting chickens when they hatch: Timing and the effects of aid on growth. Econ J.

[25] Vergara R (2010). Taxation and private investment: Evidence for Chile. Appl Econ.

[26] Que J, Lu L, Shi L. Development and challenges of mental health in China. Gen Psychiatry. 2019;32(1). 10.1136/gpsych-2019-100053 PMC6551437 31179426

[27] Missinne S, Vandeviver C, Van de Velde S, Bracke P (2014). Measurement equivalence of the CES-D 8 depression-scale among the ageing population in eleven European countries. Soc Sci Res.

[28] Schane RE, Woodruff PG, Dinno A, Covinsky KE, Walter LC (2008). Prevalence and risk factors for depressive symptoms in persons with chronic obstructive pulmonary disease. J Gen Intern Med.

[29] Shojaee M, French CJP (2014). The relationship between mental health components and locus of control in youth.

[30] Heaslip V, Vahdaninia M, Hind M, Darvill T, Staelens Y, O'Donoghue D, Drysdale L, Lunt S, Hogg C, Allfrey M (2020). Locating oneself in the past to influence the present: Impacts of Neolithic landscapes on mental health well-being. Health Place.

[31] Abdel-Khalek A, Lester D. Mental health, subjective well-being, and religiosity: Significant associations in Kuwait and USA. J Muslim Mental Health. 2013;7(2).

[32] Zhai X: Guanxi or social capital. Society (in Chinese) 2009(01):114-126+231.

[33] Bian Y (2004). Source and function s of urbanites' social capital: a network approach. Soc Sci China.

[34] Hudik M, Fang ES (2020). Money or in-kind gift? Evidence from red packets in China. J Institution Econ.

[35] Yan Y (1996). The culture of guanxi in a North China village. China J.

[36] Kipnis AB (1996). The language of gifts: Managing guanxi in a North China village. Modern China.

[37] Taber KS (2018). The use of Cronbach’s alpha when developing and reporting research instruments in science education. Res Sci Educ.

[38] Van Griethuijsen RA, van Eijck MW, Haste H, den Brok PJ, Skinner NC, Mansour N, Gencer AS, BouJaoude S (2015). Global patterns in students’ views of science and interest in science. Res Sci Educ.

[39] Cao D, Zhou Z, Si Y, Xiao X, Wang X, Shen C, Ren Y, Su M, He S, Gao J (2019). Prevalence and income-related equity in hypertension in rural China from 1991 to 2011: differences between self-reported and tested measures. BMC Health Serv Res.

[40] Wu Y (2006). Overweight and obesity in China. Bmj..

[41] Bei-Fan Z, China CMaGoWGoOi: Predictive values of body mass index and waist circumference for risk factors of certain related diseases in Chinese adults: study on optimal cut-off points of body mass index and waist circumference in Chinese adults. In.: Wiley Online Library; 2002. 12046553

[42] Kong ST, Wu Q (2019). Chinese family and society dynamics using the china family panel studies (CFPS) household panel. Aus Econ Rev.

[43] Wang D, Sun G: Research on Influencing Factors of Tourism Consumption of Urban Families in China. Age 2019, 3:1.563.

[44] Cao W, Li L, Zhou X, Zhou C (2015). Social capital and depression: evidence from urban elderly in China. Aging Ment Health.

[45] Han K-M, Han C, Shin C, Jee H-J, An H, Yoon H-K, Ko Y-H, Kim S-H (2018). Social capital, socioeconomic status, and depression in community-living elderly. J Psychiatr Res.

[46] Helliwell JF, Putnam RD (2004). The social context of well–being. Philos Trans R Soc Lond B Biol Sci.

[47] Rodríguez-Pose A, Von Berlepsch V (2014). Social capital and individual happiness in Europe. J Happiness Stud.

[48] Malinowski B (2013). Argonauts of the western Pacific: An account of native enterprise and adventure in the archipelagoes of Melanesian.

[49] Fei X: Rural China and birth system (in Chinese); 1998.

[50] Carlisle E, Flynn D (2005). Small business survival in China: Guanxi, legitimacy, and social capital. J Dev Entrepreneur.

[51] Xiao T (2006). Clan and village governance in today’ s China. J Literature History Philo (in Chinese).

[52] Chen X (2014). Gift-giving and network structure in rural china: Utilizing long-term spontaneous gift records. PLoS One.

[53] Atuoye KN, Luginaah I (2017). Food as a social determinant of mental health among household heads in the Upper West Region of Ghana. Soc Sci Med.

[54] Kim HS, Kwon M, Lee J (2016). Job stress and mental health of female household head workers. Kor J Occupational Health Nurs.

[55] Pearson JA, Geronimus AT (2011). Race/ethnicity, socioeconomic characteristics, coethnic social ties, and health: evidence from the national Jewish population survey. Am J Public Health.

[56] Vincens N, Emmelin M, Stafström M (2018). Social capital, income inequality and the social gradient in self-rated health in Latin America: A fixed effects analysis. Soc Sci Med.

[57] Sun X, Rehnberg C, Meng Q (2009). How are individual-level social capital and poverty associated with health equity? A study from two Chinese cities. Int J Equity Health.

[58] Sun J, Zhou Q (2017). Analysis on the relationship of mental health, income and income inequality among Chiese mid-aged and elderly. Chinas Prices (in Chinese).

[59] Wahlbeck K, Cresswell-Smith J, Haaramo P, Parkkonen J (2017). Interventions to mitigate the effects of poverty and inequality on mental health. Soc Psychiatry Psychiatr Epidemiol.

[60] Ridley M, Rao G, Schilbach F, Patel V: Poverty, Depression, and Anxiety: Causal Evidence and Mechanisms. Social Science Electronic Publishing 10.1126/science.aay0214 33303583

[61] Jorge A, Molina AJ, Fernández-Villa T, Artazcoz L, Martín V. Mental health, family roles and employment status inside and outside the household in Spain. Gac Sanit. 2018;S0213911118300013. 10.1016/j.gaceta.2017.11.005 29452752

[62] Velten J, Bieda A, Scholten S, Wannemüller A, Margraf J (2018). Lifestyle choices and mental health: a longitudinal survey with German and Chinese students. BMC Public Health.

[63] Larsson U, Karlsson J, Sullivan M: Impact of overweight and obesity on health-related quality of life--a Swedish population study. Int J Obes Relat Metab Disord 2002, 26(3):417-424. 10.1038/sj.ijo.0801919 11896499

[64] McLaren L, Beck CA, Patten SB, Fick GH, Adair CE (2008). The relationship between body mass index and mental health. Soc Psychiatry Psychiatr Epidemiol.

[65] Zhu Y, Qi W, Pang G, Lin L, Origasa H, Wang Y, Jie D, Mai S, Fan C, Shi H (2015). Association between Body Mass Index and Health-Related Quality of Life: The "Obesity Paradox" in 21,218 Adults of the Chinese General Population. Plos One.

[66] Park SJ, Kim SY, Lee E-S, Park S (2020). Associations among employment status, health behaviors, and mental health in a representative sample of South Koreans. Int J Environ Res Public Health.

