# Correction: Does social capital buffer or exacerbate mental health inequality? Evidence from the China Family Panel Study (CFPS)

**DOI:** 10.1186/s12939-022-01690-9

**Published:** 2022-07-12

**Authors:** Dan Cao, Zhongliang Zhou, Guanping Liu, Chi Shen, Yangling Ren, Dantong Zhao, Yaxin Zhao, Qiwei Deng, Xiaohui Zhai

**Affiliations:** 1 School of Public Policy and Administration, Xi’an Jiaotong University, No. 28 Xianning West Road, Xi’an, Shaanxi PR China; 2 School of Public Administration, Southwestern University of Finance and Economics, Xi’an, PR China; 3 School of Public Health, Xi’an Jiaotong University, Xi’an, PR China

**Correction: Int J Equity Health 21, 75 (2022)**

**https://doi.org/10.1186/s12939-022-01642-3**

After publication of this article [1], the authors reported that the legend for Fig. 3 was incorrectly given as ‘Distribution of mental health indicators’, and should have read ‘Distribution of SCs’.

Moreover, In Fig. 2(a), a superfluous symbol appeared on the right-hand side (‘The Richest’); the figure should have appeared as shown below.

**Fig. 2:**
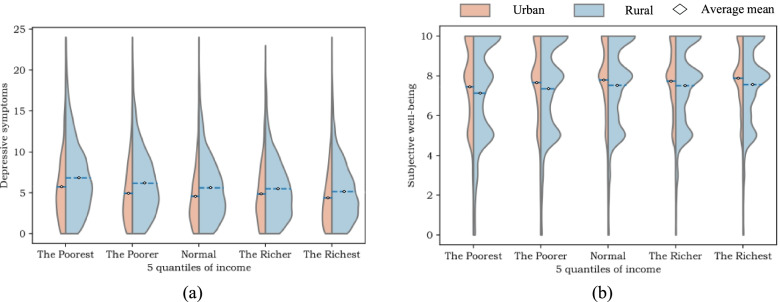
Distribution of mental health indicators

**Fig. 3:**
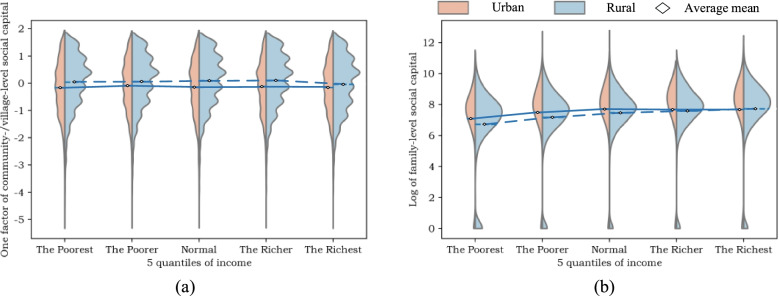
Distribution of SCs

The original article [1] has been updated.
