# Supplementary material for: Does social capital buffer or exacerbate mental health inequality? Evidence from the China Family Panel Study (CFPS)
Source: Int J Equity Health. 2022 May 23;21:75. doi: 10.1186/s12939-022-01642-3 (PMC9128128; doi:10.1186/s12939-022-01642-3)
Supplement: Supplementary file 1 — Additional file 1. [file 12939_2022_1642_MOESM1_ESM.docx]

Table A1 VIF of regression models

| Variables | Depressive symptoms | | | SWB | | |
| --- | --- | --- | --- | --- | --- | --- |
|  | Total | Urban | Rural | Total | Urban | Rural |
| Family-level SC | 1.07 | 1.06 | 1.07 | 1.07 | 1.06 | 1.07 |
| Community-/Village- level SC | 1.05 | 1.05 | 1.05 | 1.05 | 1.05 | 1.05 |
| Log of income level | 1.40 | 1.24 | 1.21 | 1.40 | 1.23 | 1.21 |
| BMI group |  |  |  |  |  |  |
| Underweight | 1.07 | 1.07 | 1.08 | 1.07 | 1.07 | 1.08 |
| Overweight | 1.11 | 1.13 | 1.11 | 1.11 | 1.13 | 1.11 |
| Obese | 1.08 | 1.10 | 1.08 | 1.08 | 1.10 | 1.08 |
| Education level |  |  |  |  |  |  |
| Junior school and below | 1.93 | 3.43 | 1.68 | 1.93 | 3.40 | 1.67 |
| High school and technical secondary school | 1.81 | 3.19 | 1.50 | 1.81 | 3.17 | 1.50 |
| Junior college | 1.64 | 2.74 | 1.38 | 1.64 | 2.72 | 1.37 |
| Bachelor and above | 1.65 | 3.05 | 1.25 | 1.65 | 3.03 | 1.25 |
| Health insurance | 1.03 | 1.05 | 1.03 | 1.03 | 1.05 | 1.03 |
| Self-rated health status | 1.13 | 1.12 | 1.13 | 1.13 | 1.12 | 1.13 |
| Working | 1.21 | 1.53 | 1.12 | 1.21 | 1.53 | 1.12 |
| Marital status |  |  |  |  |  |  |
| Married | 2.37 | 2.46 | 2.40 | 2.38 | 2.47 | 2.41 |
| Divorced | 1.29 | 1.45 | 1.24 | 1.29 | 1.45 | 1.24 |
| Widowed | 2.18 | 2.18 | 2.21 | 2.20 | 2.20 | 2.23 |
| Rural | 1.42 |  |  | 1.42 |  |  |
| Male | 1.13 | 1.11 | 1.16 | 1.13 | 1.11 | 1.16 |
| Region |  |  |  |  |  |  |
| Middle | 1.49 | 2.01 | 1.37 | 1.49 | 2.01 | 1.38 |
| Eastern | 1.59 | 2.10 | 1.46 | 1.59 | 2.10 | 1.46 |
| Age | 1.86 | 2.42 | 1.79 | 1.86 | 2.42 | 1.79 |

**Concentration Index and its Decomposition**

Concentration index (CI) is widely used to evaluate health inequality[39]. In this current study, we calculated the CIs of depressive symptoms and SWB to investigate their income-related inequality. CI ranges from -1 to 1. If there is no inequality in mental health, CI equals 0. If CI is negative, it indicates that depressive symptom/SWB is pro-poor and positive, pro-rich. The equation of CI is as follow.

$$CI=\frac{2}{\mu}cov(y,R_{i})$$

Where R_i_ is the proportion of individual i in the sample sorted by income level, and y is mental health indicators, $\mu$ is the average value of mental health indicators.

After calculating the CIs of our indicators, we decomposed them to analyze the contribution of social capital using the OLS regression model. The following equation is the linear regression model indicating the mental health ($y$) is predicted by a series of determinants ($x_{k}$):

$$y=\alpha+\sum_{k} \beta_{k}x_{ki}+\varepsilon_{i}$$

Where i means individual i, and $\beta_{k}$ is the coefficient and $\varepsilon_{i}$ is the error term. Decomposition of CI can help us evaluate how much social capital contributes to mental health inequality. If the mark of the contribution of SC is opposite to the CI, SC plays a negative role in mental health inequality; otherwise, positive.

**Result**

To furtherly confirm the buffer effect social capital plays in mental health, we conducted concentration index and its decomposition to analyze mental health inequality and the contribution of SC. The concentration indexes of depressive symptoms in the urban area and SWB in the rural area are presented in table A2. For depressive symptoms, the negative CI indicates that the CES-D8 score is pro-poor. Since the CES-D8 score is an indicator of depressive symptoms, it is obvious that the poor are more likely to be depressed. The positive CI of SWB indicates that happiness in the rural area is pro-rich. These two CIs suggest mental health inequality exists in the urban and rural areas.

Table A2 Concentration indexes

|  | Concentration index | 95% Confidential interval | |
| --- | --- | --- | --- |
| Total |  |  |  |
| Depressive symptom | -0.068 | -0.074 | -0.063 |
| SWB | 0.014 | 0.012 | 0.016 |
| Urban |  |  |  |
| Depressive symptom | -0.049 | -0.060 | -0.038 |
| SWB | 0.011 | 0.007 | 0.014 |
| Rural |  |  |  |
| Depressive symptom | -0.057 | -0.063 | -0.051 |
| SWB | 0.011 | 0.009 | 0.014 |

We have concluded from the above sections that family-level SC has a buffer effect on depressive symptom inequality in the urban area, and that village-level SC has a buffer effect on SWB inequality in the rural area. In this part, we will furtherly decompose the concentration indexes of depressive symptoms in the urban area and SWB in the rural area, and observe how much family-level SC and village-level SC contribute to the inequality respectively. In table 7, we list the percentage contribution of family-level SC to urban depressive symptom inequality and village-level SC to rural SWB inequality. The contribution of family-level SC to urban depressive symptom inequality is 3.755%, which indicates that family-level SC could buffer the pro-poor inequality of depressive symptoms in the urban area. The percentage contribution of village-level SC to rural SWB inequality is -4.651%. this result similarly indicates that village-level SC provides a buffer effect to reduce the pro-rich inequality of SWB in the rural area. The contributions of other variables are shown in table A3 in the appendix.

Table A3 Percentage contribution of SCs on mental health inequality

|  | Percentage contribution (%) |
| --- | --- |
| Total |  |
| Family-level SC on depressive symptom | 2.210 |
| Family-level SC on SWB | 4.752 |
| Community-/village- level SC on depressive symptom | -3.950 |
| Community-/village- level SC on SWB | -10.098 |
| Urban |  |
| Family-level social capital on depressive symptom | 3.177 |
| Family-level social capital on SWB | 4.930 |
| Community - level SC on depressive symptom | -0.523 |
| Community - level SC on SWB | -1.046 |
| Rural |  |
| Family-level social capital on depressive symptom | -1.681 |
| Family-level social capital on SWB | 5.172 |
| Village- level SC on depressive symptom | 1.651 |
| Village- level SC on SWB | -4.651 |
